# Plasma Calcium Level and C-Reactive Protein Albumin Ratio Affect Severe Bleeding After Coronary Artery Bypass Grafting

**DOI:** 10.21470/1678-9741-2022-0378

**Published:** 2023-06-14

**Authors:** Serdar Badem, Ahmet Yuksel, Ali Onder Kilic, Atilla Pekcolaklar, Nofel Ahmet Binicier, Demir Cetintas, Mehmet Coskun, Haluk Mevre Ozgoz, Yusuf Velioglu

**Affiliations:** 1 Cardiovascular Surgery Department, Bursa City Hospital, Bursa, Turkey; 2 Department of Thoracic Surgery, Bursa City Hospital, Bursa, Turkey; 3 Cardiovascular Surgery Department, Yalova State Hospital, Bursa, Turkey

**Keywords:** Calcium, Coronary Artery Bypass, Chest Tubes, Albumins, Drainage, Hematologic Diseases, C-Reactive Protein

## Abstract

**Objective:**

In this study, we aimed to determine whether plasma calcium level and C-reactive protein albumin ratio (CAR) as well as other demographic and hematological markers are related in predicting severe bleeding after coronary artery bypass grafting (CABG).

**Methods:**

A total of 227 adult patients who underwent CABG at our hospital between December 2021 and June 2022 were prospectively studied. Total amount of chest tube drainage was evaluated within the first 24 hours postoperatively or until the patient was re-explored for bleeding. The patients were divided into two groups - Group 1, patients with low amount of bleeding (n=174), and Group 2, patients with severe bleeding (n=53). Univariate and multivariate regression analyzes were performed to determine independent parameters related to severe bleeding within the first 24 hours after surgery.

**Results:**

When the groups were compared in terms of demographic, clinical, and preoperative blood parameters; cardiopulmonary bypass time and serum C-reactive protein (CRP) levels were found to be significantly higher in Group 2 compared to the low bleeding group. In addition, lymphocytes, hemoglobin, calcium, albumin, and CAR were found to be significantly lower in Group 2. In multivariate analysis, calcium, albumin, CRP, and CAR were found to be independent predictors of significant association with excessive bleeding. A cut-off value of 8.7 (94.3% sensitivity and 94.8% specificity) for calcium and 0.155 (75.4% sensitivity and 80.4% specificity) for CAR predicted excessive bleeding.

**Conclusion:**

Plasma calcium level, CRP, albumin, and CAR can be used to predict severe bleeding after CABG.

**Table:** 

Abbreviations, Acronyms & Symbols				
ACT	= Activated coagulation time		FFP	= Fresh frozen plasma
AF	= Atrial fibrillation		HT	= Hypertension
aPTT	= Activated partial thromboplastin time		ICU	= Intensive care unit
AUC	= Area under the curve		IQR	= Interquartile range
BMI	= Body mass index		LIMA	= Left internal mammary artery
CABG	= Coronary artery bypass grafting		MPV	= Mean platelet volume
CAR	= C-reactive protein albumin ratio		NPV	= Negative predictive value
CI	= Confidence interval		OR	= Odds ratio
COPD	= Chronic obstructive pulmonary disease		PCT	= Platecrit
CPB	= Cardiopulmonary bypass		PDW	= Platelet distribution width
CRF	= Chronic kidney failure		PPV	= Positive predictive value
CRP	= C-reactive protein		PT	= Prothrombin time
DM	= Diabetes mellitus		RDW-SD	= Erythrocyte distribution width - standard deviation
EF	= Ejection fraction		ROC	= Receiver operating characteristic
ES	= Erythrocyte suspension		WBC	= White blood cell

## INTRODUCTION

Bleeding is a common and serious complication following coronary artery bypass grafting (CABG). Patients may enter a clinical picture compatible with hypovolemic and cardiogenic shock due to excessive blood loss. Some patients with postoperative bleeding need reoperation due to the development of cardiac tamponade. In addition, bleeding diathesis and disseminated intravascular coagulation secondary to excessive use of blood products may be encountered. In addition to these, problems such as the need for prolonged mechanical ventilator support, increased hospital infections, renal dysfunction, and prolonged intensive care unit (ICU) and hospital stay may occur^[1]^. Therefore, predicting postoperative bleeding and taking necessary precautions will reduce morbidity and mortality rates and, also importantly, reduce hospital costs.

Recently, studies on the creation and use of more suitable protocols for improving hemostasis and reducing the use of blood products have gained popularity^[2]^. Numerous risk factors have been described in the literature that predict postoperative bleeding after cardiac surgery. Preoperative use of antithrombotic agents and their cut-off times, some hematological diseases, comorbid diseases (diabetes mellitus [DM], hypertension [HT], chronic obstructive pulmonary disease [COPD], chronic kidney failure [CRF], etc.), new techniques, and equipment for cardiopulmonary bypass (CPB) are some of them^[3,4]^. In addition, the WILL-BLEED Risk Scoring system was created using these risk factors to predict severe bleeding after CABG^[5]^.

In this study, we aimed to determine the relationship between plasma calcium level and C-reactive protein albumin ratio (CAR) and other demographic, clinical, and hematological markers in predicting severe bleeding after CABG.

## METHODS

### Ethical Issues

The approval for the study was obtained from the ethics committee of our hospital (Approval no: 2021-21/7, date: 17.11.2021). The study was conducted in accordance with tenets of the Declaration of Helsinki. All patients included in the study were given detailed information about the study and the operation, and their verbal and written consents were obtained.

### Study Population and Design

We prospectively evaluated 227 adult patients who underwent elective isolated CABG under CPB between December 2021 and June 2022 in our hospital, and these patients consisted of our study population. The total amount of chest tube drainage was monitored for the first 24 hours postoperatively or until the patient was reoperated for bleeding. Patients were divided into two groups according to the amount of chest tube drainage - Group 1, patients with low bleeding (n=174, 76.7%, amount of drainage < 1000 ml/24 hours), and Group 2, patients with excessive bleeding (n=53, 23.3%, amount of drainage ≥ 1000 ml/24 hours). The groups were compared in terms of demographic, clinical, and preoperative blood parameters. Preoperative demographic data (age, sex, weight, body mass index [BMI]), comorbid factors (DM, COPD, HT, CRF), and smoking status were recorded. In the preoperative period, patients’ complete blood count parameters (including hemoglobin, hematocrit, white blood cell, platelet, neutrophil, and lymphocyte), coagulation parameters (including prothrombin time [PT], partial PT, and fibrinogen) as well as serum C-reactive protein (CRP), albumin, and calcium levels, and ejection fraction values were recorded. Intraoperative CPB time, cross-clamping time, and the number of bypass grafts were also recorded.

The exclusion criteria were as follows: emergency surgery (it could be associated with excessive bleeding due to antiaggregant medications), reoperation, concomitant operations such as CABG + mitral valve surgery, less than three-vessel CABG (it could be associated with lower CPB times), malignancy, sepsis, severe hepatic and renal failures, autoimmune and hematological diseases, and other medical conditions that predispose to bleeding.

### Surgical Approach

All patients were operated through standard median sternotomy under general anesthesia. Pediculated left internal mammary artery (LIMA) and great saphenous vein were prepared with standard fashion and utilized as bypass grafts. Monopolar electrocautery was used during pediculated LIMA preparation. Before standard cannulation, 350 units/kg of unfractionated heparin were administered. When the activated coagulation time (ACT) was > 400 seconds, aortic and right atrial two-stage venous cannulation was performed and CPB was entered. During CPB, in the nonpulsatile phase, 2-2.5 l/min/m^2^ flow rate, 50-70 mmHg mean arterial blood pressure, and 20-25% hematocrit levels were achieved. With the end-to-side anastomosis technique, first the distal anastomoses were performed using 7/0 PROLENE®, and then the proximal anastomoses were performed using 6/0 PROLENE®. In order to minimize reperfusion injury, hot blood (hot shot) was given just before the cross-clamp was removed. When suitable conditions were established in the patients, CPB was discontinued and decannulated. To neutralize the effect of heparin, 1-1.3 mg of protamine sulfate per 1 mg of heparin was administered, and the ACT value was brought to normal limits. After bleeding control, the tissues were duly closed, and operation was terminated. Operations were performed on all patients by the same surgical team. All patients were transferred to the ICU immediately after the operation.

### Intensive Care Unit Follow-up

During the early postoperative period, invasive arterial blood pressure, central venous pressure, heart rhythm, oxygen saturation, urine output, and mediastinal drainage were continuously monitored. Arterial blood gas analysis and electrolyte monitoring were performed every hour for the first six hours. In addition, the amount of bleeding in the first 24 hours after the operation was calculated and recorded in milliliters. ACT was measured every hour, and an additional dose of protamine sulfate was administered when it was > 120 seconds. Perioperative erythrocyte suspension (ES) and fresh frozen plasma (FFP) usage, lengths of ICU and hospital stays, complications, and mortality rates were recorded. In our daily clinical practice, ES transfusion is not performed until the hematocrit level decreases < 25%. Our criteria for reoperation due to bleeding are as follows^[6]^:

1. Bleeding > 400 ml/hour in the first postoperative hour.
2. Bleeding > 300 ml/hour in the second and third hours.
3. Bleeding > 200 ml/hour for the first four hours.
4. Presence of cardiac tamponade.
5. Immediate drainage with low hematocrit levels.

### Statistical Analysis

**Fig. 1:**
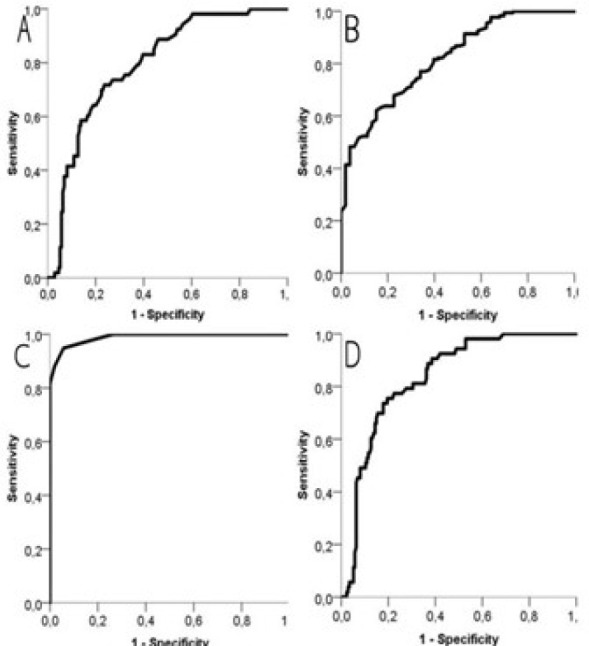
Receiver operating characteristic curve analysis for parameters identified as independent risk factors in multivariate analysis.

Data were entered into the Statistical Package for the Social Sciences (IBM® SPSS Statistics for Windows, Version 23.0, Armonk, New York, United States of America) software package. Descriptive statistics were used, and quantitative variables were characterized using mean, maximum (max), and minimum (min) values; percentages were used for qualitative variables. Whether the distributions were normal or not was determined by Kolmogorov-Smirnov analysis. Normal distributions were reported as mean values. Student’s *t*-test was used for comparisons between groups. Pearson’s Chi-square test was used for comparative analysis of qualitative variables; however, Fisher’s exact test was used if the sample size was small (≤ 5). Nonparametric continuous variables were recorded as medians and compared using Mann-Whitney U tests. Interquartile range results were also given for the values recorded as median. *P*-value < 0.05 was considered statistically significant. Multivariate analysis was performed using the variables found to affect the drainage > 1000 ml in the univariate analysis. In the multivariate analysis, the area under the curve (AUC) was calculated by performing receiver operating characteristic (ROC) curve analysis for the nonparametric independent risk factors that were found to affect the drainage > 1000 ml (Figure 1). In addition, the best sensitivity and specificity values were accepted as the cut-off values.

## RESULTS

There were statistically significant differences between the groups with regards to CPB time, lymphocyte count, CRP, hemoglobin, calcium, albumin, and CAR. CPB time was longer in Group 2 than in Group 1. Lymphocyte count, hemoglobin, calcium, and albumin levels were higher in Group 1 compared to Group 2, while CRP levels and CAR were lower. The comparison between the groups in terms of demographic, clinical, and preoperative blood parameters is given in Table 1.

**Table 1:** Grouping of patients according to the amount of drainage and comparison between the groups.

Variable	Group 1 (n=174)	Group 2 (n=53)	*P*-value
Age (years), median (IQR)	61 (13)	62 (11)	0.225
Sex, n (%)			
Male	133 (76.4)	35 (66.0)	0.131
Female	41 (23.6)	18 (34.0)	
BMI (kg/m^2^), median (IQR)	26.9 (4.7)	26.6 (5.0)	0.368
DM, n (%)	79 (45.4)	21 (39.6)	0.458
HT, n (%)	79 (45.4)	24 (45.3)	0.988
COPD, n (%)	9 (5.2)	4 (7.5)	0.515
Smoker, n (%)	56 (32.2)	18 (34.0)	0.809
CABG graft count, median (IQR)	4 (1)	4 (1)	0.716
EF (%), median (IQR)	55 (15)	50 (15)	0.678
CPB time (min), median (IQR)	76.5 (35.3)	88.0 (38.5)	0.003
Cross-clamp time (min), median (IQR)	56.0 (21.0)	63.0 (26.5)	0.098
WBC (10^3^/µL), median (IQR)	8.6 (3.3)	8.3 (2.6)	0.214
Platelet (10^3^/µL), median (IQR)	250.0 (99.8)	265.5 (102.8)	0.494
Neutrophil (10^3^/µL), median (IQR)	5.4 (3.0)	5.3 (2.6)	0.703
Lymphocyte (10^3^/µL), median (IQR)	2.2 (0.9)	1.8 (1.0)	0.007
PCT (%), median (IQR)	0.26 (0.10)	0.27 (0.11)	0.654
MPV (fL), median (IQR)	10.3 (1.4)	10.2 (1.5)	0.506
PDW (fL), median (IQR)	11.7 (3.4)	11.5 (3.3)	0.313
RDW-SD (fL), median (IQR)	40.1 (4.4)	39.8 (5.7)	0.665
CRP (mg/L), median (IQR)	3.1 (3.7)	8.6 (8.1)	< 0.001
Hemoglobin (g/dL), median (IQR)	14.1 (2.5)	12.7 (2.8)	< 0.001
PT (s), median (IQR)	8.8 (0.8)	8.9 (1.0)	0.113
aPTT (s), median (IQR)	29.2 (5.6)	30.7 (5.9)	0.216
Fibrinogen (mg/dL) median (IQR)	442.5 (180.5)	455.0 (246.3)	0.094
Calcium (mg/dL), median (IQR)	9.4 (0.5)	8.3 (0.5)	< 0.001
Albumin (g/L), median (IQR)	42.1 (5.1)	38.3 (7.4)	< 0.001
CAR, median (IQR)	0.07 (0.08)	0.23 (0.18)	< 0.001
ES, median (IQR)	3 (3)	6 (1.5)	< 0.001
FFP, median (IQR)	4 (2)	4 (3)	0.005
AF, n (%)	21 (12.1)	12 (22.6)	0.05
Bleeding revision, n (%)	2 (1.1)	6 (11.3)	0.002
ICU stay	3 (2)	3 (1)	0.007
Hospital stay	7 (2)	8 (2.5)	0.02
Mortality n (%)	1 (0.6)	4 (7.5)	0.01

Bold *P*-values indicate statistical significance; italicized *P*-values are values close to statistical significance

AF=atrial fibrillation; aPTT=activated partial thromboplastin time; BMI=body mass index; CABG=coronary artery bypass grafting; CAR=C-reactive protein albumin ratio; COPD=chronic obstructive pulmonary disease; CPB=cardiopulmonary bypass; CRP=C-reactive protein; DM=diabetes mellitus; EF=ejection fraction; ES=erythrocyte suspension; FFP=fresh frozen plasma; HT=hypertension; ICU=intensive care unit; IQR=interquartile range; MPV=mean platelet volume; PCT=platecrit; PDW=platelet distribution width; PT=prothrombin time; RDW-SD=erythrocyte distribution width - standard deviation; WBC=white blood cell

According to the multivariate analysis, the identified independent risk factors affecting postoperative severe bleeding were as follows: CRP (odds ratio [OR]=1.217, 95% confidence interval [CI]=1.020-1.452, *P*=0.02), calcium (OR=0.112, 95% CI=0.099-0.231, *P*<0.001), albumin (OR=0.695, 95% CI=0.497-0.972, *P*=0.03), and CAR (OR=831.0, 95% CI=5.844-11817.157), *P*=0.008) (Table 2). Afterwards, ROC curve analyzes were also performed to determine the optimum threshold values of the identified independent risk factors with sensitivity and specificity rates. It was observed that the parameter with the best AUC value was calcium (AUC=0.989, 95% CI=0.965-0.998). Threshold values were determined according to the best sensitivity and specificity values for all parameters (Table 3). Accordingly, patients were divided into groups with low and high values (Table 4).

**Table 2:** Investigation of independent risk factors affecting drainage > 1000 ml by multivariate logistic regression analysis.

Variable	Multivariate analysis^1^			Multivariable analysis^2^		
	Odds ratio	95% CI	*P*-value	Odds ratio	95% CI	*P*-value
CPB time	1.009	0.987-1.030	0.436	1.020	0.997-1.043	0.08
Lymphocyte	1.976	0.661-5.905	0.223	1.320	0.551-3164	0.533
CRP	1.217	1.020-1.452	0.02	--	--	--
Hemoglobin	0.607	0.286-1.286	0.192	0.607	0.335-1.100	0.100
Calcium	0.012	0.001-0.099	< 0.001	0.011	0.002-0.100	< 0.001
Albumin	0.695	0.497-0.972	0.03	--	--	--
CAR	--	--	--	831.0	5.844-11817.157	0.008

Bold *P*-values indicate statistical significance

1 Multivariate analysis includes CRP and albumin

2 Multivariable analysis includes CAR, not CRP and albumin

CAR=C-reactive protein albumin ratio; CI=confidence interval; CPB=cardiopulmonary bypass; CRP=C-reactive protein

**Table 3:** ROC curve analysis for parameters determined as independent risk factors affecting drainage > 1000 ml in multivariate analysis.

Variable	AUC	95% CI for AUC	Threshold value	Sensitivity (%)	Specificity (%)	PPV (%)	NPV (%)
CRP	0.795	0.736-0.845	5.6	71.7	76.4	48.1	89.9
Calcium	0.989	0.965-0.998	8.7	94.3	94.8	84.7	98.2
Albumin	0.818	0.761-0.866	41.2	84.9	62.0	40.5	93.1
CAR	0.835	0.780-0.880	0.155	75.4	80.4	54.1	91.5

AUC=area under the curve; CAR=C-reactive protein albumin ratio; CI=confidence interval; CRP=C-reactive protein; NPV=negative predictive value; PPV=positive predictive value

**Table 4:** Grouping of patients according to the determined threshold values and comparison between these groups according to whether the drainage is > or < 1000 ml.

Variable	Threshold value	Is the drainage > 1000 ml?		*P*-value	OR	95% CI for OR
		No (n=174)	Yes (n=53)			
CRP	≤ 5.6	133 (76.4%)	15 (28.3%)	< 0.001	8.218	4.111-16.428
	> 5.6	41 (23.6%)	38 (71.7%)			
Calcium	≤ 8.7	9 (5.2%)	50 (94.3%)	< 0.001	0.003	0.001-0.013
	> 8.7	165 (94.8%)	3 (5.7%)			
Albumin	≤ 41.2	66 (37.9%)	45 (84.9%)	< 0.001	0.109	0.048-0.245
	> 41.2	108 (62.1%)	8 (15.1%)			
CAR	≤ 0.155	140 (80.5%)	13 (24.5%)	< 0.001	12.67	6.110-26.274
	> 0.155	34 (19.5%)	40 (75.5%)			

CAR=C-reactive protein albumin ratio; CI=confidence interval; CRP=C-reactive protein; OR=odds ratio

Group 1 patients were found to have statistically lower CRP values compared to Group 2 patients (OR=8.218, 95% CI=4.111-16.428, *P*<0.001). Group 2 patients were found to have lower calcium levels at a statistically higher rate than Group 1 patients (OR=0.003, 95% CI=0.001-0.013, *P*<0.001). Similarly, Group 1 patients were found to have albumin levels statistically higher than Group 2 patients (OR=0.109, 95% CI=0.048-0.245, *P*<0.001). Group 1 patients were found to have statistically lower CAR values than Group 2 patients (OR=12.670, 95% CI=6.110-26.274, *P*<0.001).

The comparison of the groups in terms of postoperative follow-up is given in Table 5. Statistically, Group 2 patients received more ES (*P*<0.001) and FFP (*P*=0.005) transfusions. Group 2 patients underwent more revisions (*P*=0.002) and also had longer ICU and hospital stays (*P*=0.007 and *P*=0.02, respectively). Statistically lower mortality was observed in Group 1 compared to Group 2 (*P*=0.01).

**Table 5:** Comparison between the groups in terms of postoperative follow-up.

Variable	Group 1 (n=174)	Group 2 (n=53)	*P*-value
ES (unit), median (IQR)	3 (3)	6 (1.5)	< 0.001
FFP (unit), median (IQR)	4 (2)	4 (3)	0.005
AF, n (%)	21 (12.1)	12 (22.6)	0.05
Bleeding revision, n (%)	2 (1.1)	6 (11.3)	0.002
ICU stay	3 (2)	3 (1)	0.007
Hospital stay	7 (2)	8 (2.5)	0.02
Mortality n (%)	1 (0.6)	4 (7.5)	0.01

Bold *P*-values indicate statistical significance

AF=atrial fibrillation; ES=erythrocyte suspension; FFP=fresh frozen plasma; ICU=intensive care unit

## DISCUSSION

In this study, considering the hematological and biochemical parameters, the median lymphocyte, hemoglobin, calcium, and albumin values were found to be lower in the group with severe bleeding compared to the other group. In addition, the median CRP and CAR values were found to be higher in the group with severe bleeding compared to the other group. In multivariate analysis, CAR, CRP, calcium, and albumin values were found to be statistically significant, and it was concluded that these parameters independently predicted severe bleeding after CABG.

There are many factors affecting postoperative bleeding in open heart surgery. Hematological diseases, pharmacological agents used preoperatively, antiaggregant agents used perioperatively, and open heart surgery using CPB are some of them^[3,4]^. Preoperative use of acetylsalicylic acid, clopidogrel, and other drugs may affect hemostatic functions and increase postoperative bleeding^[7,8]^. In our study, aspirin and clopidogrel were discontinued seven days before surgery and warfarin five days before surgery in all patients. Low-molecular-weight heparin treatment was stopped 12 hours before surgery. Widespread microvascular bleeding may develop due to the use of CPB in coronary artery surgery. The use of heparin, development of systemic inflammation, and negative effects of hypothermia on the hemostatic system (decreased platelet number and dysfunction, fibrinogen level, and coagulation factor consumption, etc.) cause this situation^[3,9,10]^. Contact of blood with CPB circuits induces coagulation activation. Thus, it causes more consumption of clotting factors and platelets in the circulation. In addition, while the crystalloid fluids used to expand volume in the CPB process cause dilution of clotting factors and platelets, the use of colloid fluids both inhibit platelet function and cause thrombocytopenia. As a result, it causes an increase in the amount of postoperative bleeding^[9,11]^.

Various risk factors associated with postoperative excessive bleeding following adult cardiac surgery were identified in a current integrative review study by Lopes et al^[3]^. In that study, reviewing a total of 17 studies from seven databases, the predictors of severe bleeding after open heart surgery were classified as patient-related, procedure-related, and postoperative factors. Patient-related factors included male sex, DM, low BMI and left ventricular ejection fraction, high preoperative hemoglobin level, low preoperative platelet counts, and fibrinogen concentration, whereas perioperative-related factors included operating surgeon, CABG with three or more grafts, internal thoracic artery usage, increased cross-clamping, CPB, and total operation times, low intraoperative body temperature, and postoperative fibrinogen levels and metabolic acidosis. The authors consequently deduced that the mentioned predictors could be utilized for risk stratification of severe bleeding following open-heart surgery, and the evaluation of patients could be guided by knowing these factors, hence perioperative awareness can be prioritized. In addition, it was also expressed that timely determination and correction of the modifiable factors could be facilitated. With reference to this review study, only increased CPB time was identified as a common risk factor in both their and our studies. Furthermore, low preoperative hemoglobin level was defined as a risk factor in our study, whereas in the review study, on the contrary, high preoperative hemoglobin level was surprisingly expressed as a risk factor for severe bleeding after cardiac surgery.

Calcium plays an important role in the platelet aggregation and coagulation cascade. Thus, it takes part in ensuring hemostasis. Additionally, calcium is a cofactor in the enzymatic system, has an impact on the control of vasomotor tone, and has significant effects on the contractility of cardiac and striated muscle^[12,13]^. In addition, *in vitro* studies have shown that there is a relationship between ionized calcium level and clot strength and concentration^[14]^. Some studies have been carried out investigating the relationship between hypocalcemia and bleeding complications in different patient groups. In a systematic review by Vasudeva et al.^[13]^, it was reported that the amount of bleeding may be excessive because of coagulopathy developing due to hypocalcemia in adult patients with multitrauma, and this situation causes an increase in mortality due to massive blood transfusion. Epstein et al.^[15]^ showed that low calcium level in the postpartum period causes an increase in the amount of postpartum hemorrhage. In another study, they reported that low serum calcium level increased the size of hematoma in patients with intracerebral hemorrhage and therefore was associated with coagulopathy^[16]^. In the ROC curve analysis of our study, we found that the parameter with the highest sensitivity and specificity in predicting severe postoperative bleeding was serum calcium level. Therefore, preoperative determination of serum calcium level may be important in predicting bleeding complications and taking necessary precautions.

CAR is developed as a novel sensitive marker that reflects the immune status of patients. Currently, predictive values of CAR have been widely studied in a wide range of diseases. Moreover, predictive values of CAR have also been examined for patients undergoing cardiac surgery. Karabacak et al.^[17]^ and Aksoy et al.^[18]^, in their studies to predict new-onset atrial fibrillation after CABG, showed that CAR indicates a higher inflammatory state and is better than CRP and albumin alone at detecting postoperative atrial fibrillation. Furthermore, CAR has been shown to be an independent predictor of saphenous vein graft disease and to have an association with atherosclerosis^[19,20]^. Kahraman et al.^[21]^ described that CAR is strongly associated with rehospitalization rates, paravalvular leak, and increased mortality rates in patients undergoing aortic valve replacement due to isolated degenerative severe aortic stenosis. To the best of our knowledge, our study was the first to determine that CAR predicted severe postoperative bleeding in patients who underwent CABG, and there is no published research on the relationship between CAR and postoperative bleeding in the literature.

Albumin is a negative acute phase reactant that is inversely related to inflammation and oxidative stress. Hypoalbuminemia is associated with the occurrence of certain cardiovascular diseases. In patients with low albumin levels before CABG surgery, postoperative atrial fibrillation, acute kidney injury, slower recovery, readmission, and mortality rates were found to be high^[17,22]^. Franco et al.^[23]^ showed that hypoalbuminemia is frequently seen after cardiac surgery, and low serum albumin levels increase the rates of sepsis, prolonged ICU stay, and in-hospital mortality due to bleeding complications. In addition, low serum albumin level is associated with endothelial damage, increased blood viscosity, and coronary artery narrowing due to platelet aggregation^[24]^. As far as we know, our study was the first to determine that low albumin levels in the preoperative period predicted postoperative bleeding.

CRP is a major acute phase reactant that rises acutely and rapidly in stress, infection, and tissue damage. There are literature studies showing that it is predictive in many diseases. In a meta-analysis including 26 studies examining the prognostic value of coronary artery disease, high CRP level was reported to be an independent predictor of major adverse cardiovascular events, cardiovascular mortality, and all-cause mortality^[25]^. In another meta-analysis in the literature, high CRP levels were reported to be a predictor of postoperative atrial fibrillation in patients undergoing coronary artery surgery^[26]^. In our study, we determined that high CRP predicts severe postoperative bleeding in patients who underwent CABG.

### Limitations

Our study had several limitations. The major limitations were the relatively small number of patients in the groups and its single-centered design. Another major limitation was that the assessed data was restricted. For example, preoperative examination of platelet function analysis and coagulation factors, which were not included in our study, may provide a better estimation of postoperative bleeding.

## CONCLUSION

To the best of our knowledge, the present study was the first one investigating whether serum calcium level and CAR, a novel biochemical marker, predicted severe bleeding after CABG. Our study demonstrated for the first time that higher serum CRP level and CAR and lower serum calcium and albumin levels were associated with severe bleeding after CABG, and these biochemical parameters could be useful for the prediction of postoperative severe bleeding following CABG. Further large-scale well-designed studies are required to support our findings and to obtain stronger scientific evidence.

**Table:** 

Authors’ Roles & Responsibilities	
SB	Substantial contributions to the conception or design of the work; or the acquisition, analysis, or interpretation of data for the work; drafting the work or revising it critically for important intellectual content; agreement to be accountable for all aspects of the work in ensuring that questions related to the accuracy or integrity of any part of the work are appropriately investigated and resolved; final approval of the version to be published
AY	Substantial contributions to the conception or design of the work; or the acquisition, analysis, or interpretation of data for the work; drafting the work or revising it critically for important intellectual content; agreement to be accountable for all aspects of the work in ensuring that questions related to the accuracy or integrity of any part of the work are appropriately investigated and resolved; final approval of the version to be published
AOK	Substantial contributions to the conception or design of the work; or the acquisition, analysis, or interpretation of data for the work; drafting the work or revising it critically for important intellectual content; agreement to be accountable for all aspects of the work in ensuring that questions related to the accuracy or integrity of any part of the work are appropriately investigated and resolved; final approval of the version to be published
AP	Substantial contributions to the conception or design of the work; or the acquisition, analysis, or interpretation of data for the work; drafting the work or revising it critically for important intellectual content; final approval of the version to be published
NAB	Substantial contributions to the conception or design of the work; or the acquisition, analysis, or interpretation of data for the work; drafting the work or revising it critically for important intellectual content; agreement to be accountable for all aspects of the work in ensuring that questions related to the accuracy or integrity of any part of the work are appropriately investigated and resolved; final approval of the version to be published
DÇ	Substantial contributions to the conception or design of the work; or the acquisition, analysis, or interpretation of data for the work; agreement to be accountable for all aspects of the work in ensuring that questions related to the accuracy or integrity of any part of the work are appropriately investigated and resolved; final approval of the version to be published
MC	Drafting the work or revising it critically for important intellectual content; agreement to be accountable for all aspects of the work in ensuring that questions related to the accuracy or integrity of any part of the work are appropriately investigated and resolved; final approval of the version to be published
HMO	Agreement to be accountable for all aspects of the work in ensuring that questions related to the accuracy or integrity of any part of the work are appropriately investigated and resolved; final approval of the version to be published
YV	Substantial contributions to the conception or design of the work; or the acquisition, analysis, or interpretation of data for the work; agreement to be accountable for all aspects of the work in ensuring that questions related to the accuracy or integrity of any part of the work are appropriately investigated and resolved; final approval of the version to be published
